# Diagnostic reproducibility on a digital evaluation system slide in cytology and histology in oncologic screening

**DOI:** 10.1186/1746-1596-8-S1-S18

**Published:** 2013-09-30

**Authors:** Stefania Lega, Paola Crucitti, Paola Pierotti, Roberta Rapezzi, Priscilla Sassoli de’ Bianchi, Carlo Naldoni, Arrigo Bondi

**Affiliations:** 1 Anatomia Patologica Ospedale Maggiore, Azienda USL di Bologna, Italy; 2 Direzione Generale Sanità e Politiche Sociali – Regione Emilia-Romagna, Italy

## Background

Diagnostic reproducibility and accuracy in cytology and histology are the main issues in Oncologic Screening of cervix, breast and colorectal cancer : it can be achieved by programs for quality assurance (QA). The slides set standard represents the most used method to compare diagnostic proficiency, the chance of interpreting microscopic digital photographs provided an interesting alternative to read conventional microscope slides.

The whole digital slide observed in a computer screen is a third, interesting, option to reach the goal. In fact all the information on conventional samples are transferred into a file, easily archived, catalogued, duplicated or advice for quality control, but is especially available at distance and from multiple locations simultaneously with drastic reduction of time needed to achieve proficiency test reproducibility [1].

The production of digital slides with modern scanners is relatively simple and quick. All suppliers offer services into private or public networks server in the literature [2] and software able to track scanned cases stored in comprehensive database to build large casistic archives online [3] Tools are already available for a teleconference discussion of cases with vision of cytological preparations on line [4,5]**,** educational programs with integrated digital slides are poorly developed or proficiency tests for continuing education and professional updating are easily accessible.

A project on Virtual Microscopy and Digital Pathology has been conducted in Emilia-Romagna Region (Italy) with the goal to promote quality in diagnostic cytology and histology in Screening programs by testing a different system involving pathologists and cytologists using digital slides, with a faster and reproducible program easier to manage than standard diagnostic sets and by distance for retraining of patrhologists with a final consensus meeting.

The aim has been reached with the realization of a management system for cytological and histological whole-slides digital images and related clinical data and the building of a picture archive and communication system (PACS) among pathologists of our (and probably other) region. This must be backed by software for the realization of network slide seminars to perform periodic diagnostic reproducibility and proficiency test. The cases, collected and properly catalogued in an online, easily accessible and systemic digital archive of slides, with diagnoses discussed in clinical and pathologic audit meetings and validated by experts, can be used as diagnostic reference tools (case registry online ). The cataloguing and indexing is performed with NAP codes derived from SNOMED [6], which contains terms and definitions in Italian and English and encompasses extensive synonyms and complex researches.

## Material and methods

**Figure 1:**
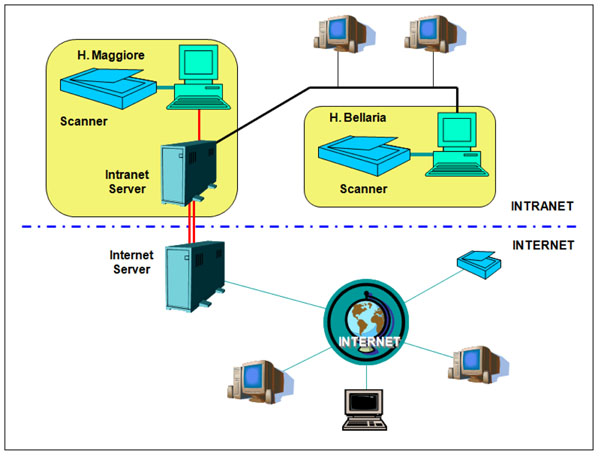
Network scheme: two scanner are in an intranet environment with an local disk server, connected to an internet server where the public products are stored.

The cancer screening group of the Emilia-Romagna Region (Italy) set up a picture, archive and communication system (PACS) devoted to pathologists for cooperative diagnosis, teaching and training, teleconsulting, documentation of rare cases and pilot experiences; furthermore selected cases are catalogued in the PACS with the aim to check the diagnostic concordance in regional oncologic screening (cervix, breast and colon). The PACS system is made by two Aperio scanner and an adequate internet server where the described programs perform, (see Figure 1) [7].

The slides have been digitalized using an Aperio scanner, 20x for histology and 40x for cytology and an internet server was used to store the files, arranged into a Spectrum database (Aperio). An e-learning platform (Docebo) [8] has been used to built interface for the applicants: cases and slides were considered “teaching instruments” for the educational software (slide seminars) and appropriate questioning forms have been designed with the diagnostic occurrences of Bethesda System 2011 for cytology and CIN options for histology for the cervical cancer and of International guidelines for breast and colorectal cancer.

Nowadays the diagnostic reproducibility has been performed in colorectal and cervical cancer screening (Bologna October 2010, Bologna June 2011), and for breast cancer is ongoing (Bologna June 2012). In all three slide seminars a number of cases have been selected by a committee of pathologists working in regional anatomo-pathologic units.

Colorectal cancer screening was the first retraining slide seminar for pathologists performed with these features in our region and probably in Italy.Three regional units (Bologna, Cesena and Ferrara) were involved by sending representative histological cases of all main diagnostic occurrences to test the diagnostic reproducibility; 28 histological cases were collected. A definite and limited time interval was indicated to study slides, then a consensus conference was organized in the same day.

The second Seminar of QA to test the diagnostic reproducibility was performed in cervical cancer screening program; 30 cytological and 30 histological cases have been selected by a committee of pathologists among the cases proposed by the regional units. All main diagnostic occurrences were represented, basic clinical information and relevant follow-up information were available; the cases have been completely anonymized.

A 30 days interval was indicated to study the slides, then a consensus conference has been programmed at the end of the evaluation to present the results and discuss cases. Before the meeting each participant received a report with the gold standard diagnosis performed buy the committee and her/his diagnosis and concordance result.

## Results and discussion

15 pathologists of regional units attended the **colon-rectal QA** and the diagnostic reproducibility have been evaluated matching their results with the final gold standard diagnosis reached during the consensus conference. The observed agreement was 69% and the overall performance of the participating pathologists was assessed with a statistical analysis using Cohen’s kappa: the average value was 0.64 (substantial).

95 cytologists and 32 histopathologists have been involved in the **cervical cancer screening QA**.

The diagnostic reproducibility has been evaluated using the final diagnosis reached in the consensus conference: in 2 out of 30 cytological cases the diagnosis was different from the diagnosis of the committee, while all histologic diagnoses were in agreement. The overall performance of the participating readers is reported in table 1.

**Table 1:** Screening PAP test - distribution of agreement in diagnosis

		Agreement in homogeneous groups: final diagnoses			
		
		negative	ASCUS L SIL	ASC-H H SIL / SqCC	AGC AIS / ADC
**Reader’s diagnosis**	**negative**	**85.1**	15	6.6	2.1
		
	**ASCUS/ L SIL**	5.1	**72.4**	20.2	3.6
		
	**ASC-H/ H SIL/ SqCC**	2.9	11.9	**67.7**	14
		
	**AGC / AIS/ ADC**	2.9	0.3	5.2	**79.2**
		
	**other tumours**	3.5	0.2	0.2	0.7
		
	**no answer**	0.5	0.2	0.1	0.4

		**100%**	**100%**	**100%**	**100%**
					
	Overall observed agreement		73%		
	Cohen's Kappa		0.63	(substantial)	

## Conclusion

Whole digital slide is suitable for proficiency tests and the internet e-learning platform allows to share cases and to get the answers from participants, in a easier way than by of a set of conventional slides.

The quality of whole slides is very good, approaching optical microscopic resolution.

In a cytological environment the bias is to get a perfect focus in all parts of the slide; the wider area of slide to examine and the higher number of diagnostic classes may justify a worse agreement of the pathologists and a poorer performance (lower Cohen’s kappa) than histology.

We have produced an integrated environment that includes many of the modern aspects of digital pathology that can be shared with the PACS system in many laboratories of the region, including quality promotion and control of image interpretation in cytology and histology applied to cancer screening.
